# Optimization design and analysis of mobile pump truck frame using response surface methodology

**DOI:** 10.1371/journal.pone.0290348

**Published:** 2023-08-17

**Authors:** Liang Cheng, Hai-Bin Lin, Yu-Liang Zhang

**Affiliations:** 1 College of Mechanical Engineering & Key Laboratory of Air-driven Equipment Technology of Zhejiang Province, Quzhou University, Quzhou, China; 2 College of Mechanical and Electrical Engineering, Northeast Forestry University, Harbin, China; University of Vigo, SPAIN

## Abstract

In order to realize the lightweight design of mobile pump truck, this paper takes the frame of a certain type of mobile pump truck as the research object. The response surface method is used to carry out lightweight design of the longitudinal beam structure of the frame, and the finite element method is used to establish the finite element model to compare and analyze the optimized and original designs. The results show that the height, width and thickness of the optimized longitudinal beam section are reduced by 10mm, 11mm, and 0.8mm respectively, and the weight of the whole frame is reduced by 35.8kg. Before and after optimization, the displacement and stress changes of the frame are small in four motion situations, which meet the lightweight requirements of optimization design.

## 1 Introduction

In recent years, mobile pump trucks have been widely used in municipal emergency rescue and drainage operations. The variability of its operating environment and the urgency of operating requirements decide that the pump truck needs to be lightweight development. As the main bearing parts of the mobile pump truck, the frame of the pump truck has always been one of the main targets of lightweight. However, the force on the frame during driving is very complex, so the frame must be that it still has enough strength after lightweight design.

Surajkumar G. Kumbhar used finite element method to study the chassis frame of four-wheel bicycle, and used FFT method to analyze the modal of the frame. According to the analysis data, the chassis frame was optimized and designed, and a lighter and safer four-wheel bicycle chassis frame was obtained [1]. Masanori Honda et al. studied the optimization of the cross section shape design of automobile frame, used nonlinear finite element method and genetic algorithm to optimize the cross section shape of automobile frame, and proposed a new design criterion of cross section shape of automobile frame [2]. Tautvydas Pravilonis et al. conducted simulation analysis by combining kinematic analysis and finite element analysis, and discussed the reliability of bus frames made of different steel profiles [3]. Ren et al. used Abaqus to analyze the inherent vibration frequencies and dynamic modes of the SX360 dump truck frame, studied the stress distribution of the dump truck frame, the deflection patterns at the ends and midpoints of the frame, and the transient frame dynamics. It was found that the frame deflection and nodal vibration amplitudes varied with the frame support position [4]. Yang et al. performed multi-objective optimization of the upper plate mechanism of the battery baler machine. The overall weight was reduced by 30.85%, and the maximum equivalent force was reduced by 19.98%, which achieved the design goals of structural optimization and lightweight [5]. Zhao et al. established a finite element model of the press frame and proposed a two-stage topology optimization method to optimize the frame structure, which reduced the volume by 13.66% while ensuring the frame performance [6]. P. Satheesh Kumar Reddy et al. optimized the design based on the tensile stiffness formula and strength-to-weight ratio characteristics of the solid shaft and hollow shaft. An optimized finite element model of the transmission shaft was established in the finite element analysis software Ansys. At the same time, static, free vibration, and tension buckling analyses were conducted on the transmission shaft to ensure maximum stiffness while carrying out lightweight design [7].

Pan et al. analyzed and verified the stress of the tricycle frame under the five working conditions of high-speed driving, turning, climbing, braking and bumpy road. According to the analysis results, the lightweight design of the frame was carried out, and the weight of the improved frame was reduced by 19.1% [8]. Feng et al. combined contribution analysis methods, experimental design, grey correlation and principal component combination analysis, and the TOPSIS method to carry out multi-objective lightweight optimization of body side structures [9]. Tufan Gürkan Yılmaz et al. used low-density materials to reduce the weight of door hinges while ensuring their adequate mechanical properties [10]. Pan et al. established the nonlinear dynamic finite element model of the battery pack housing, and completed the lightweight design of the battery pack housing based on the governing equation and the explicit finite element program LS-DYNA [11]. Li et al. established the finite element model of the body-in-white, calculated the bending stiffness and torsional stiffness of the body according to the actual working conditions of the vehicle. They studied the main opening deformation of the body under bending and torsional conditions, and optimized the body structure with the minimum body weight as the optimization variable [12]. C.D. Horvath has carried out relevant research on lightweight materials for lightweight design of automobile architecture. Appropriate materials can enable components and vehicles to give full play to their best design capabilities [13]. Oguz Dogan et al. redesigned the parts using modern optimization techniques, redesigned the tractor clutch finger using topology and shape optimization methods, and carried out fatigue analysis based on stress-life. The optimal finger size was obtained by shape optimization and response surface method. Compared with the initial design, the maximum stress and mass were reduced by 14% and 6% respectively, and the stiffness was increased by 31.6% [14].

Girish Dutt Gautam et al. designed and optimized the roll cage of a formula racing car, and studied the stress and deformation of the roll cage under different loading conditions [15]. Wang et al. designed a new type of bus frame and established a finite element model using Ansys Workbench software. Numerical calculations for the load conditions of bus frames under five typical operating conditions such as full load bending and braking [16]. Chen used finite element analysis software to simulate and calculate the stress conditions of the chassis under full load bending conditions, and verified the accuracy of the simulation results through experimental methods. Further research was conducted on the stress conditions of the chassis under four typical conditions: full load bending, bending-torsion coupling, emergency braking, and emergency turning [17]. Liu et al. used HyperWorks software to conduct Statics analysis on a trailer frame, studied its stress and deformation under three load distributions and four typical working conditions, and optimized the deployment of the frame based on the analysis results [18]. Jiang et al. redesigned the longitudinal beam structure of an electric commercial vehicle frame as the research object, and used the thickness of the longitudinal beam and crossbeam of the frame as the design variables. The first order modal frequency, maximum displacement under bending conditions, and maximum displacement under torsion conditions were used as constraints. The lightweight design was carried out with the objective function of minimizing the mass of the frame [19]. Zhang et al. used the finite element software Ansys Workbench to carry out static analysis and modal analysis of a lathe spindle, and on this basis, combined the response surface method and MOGA optimization algorithm to carry out lightweight design of the spindle [20]. Saber Moradi et al. combined sensitivity analysis and response surface method to study the influence of relevant design parameters of PT materials on CRSBF seismic response [21]. Sun et al. combined single factor test with response surface method to explore the law of influence of sizing amount, hot pressing temperature and hot pressing time on static bending strength and bonding strength of composite materials, and optimized the preparation process of bamboo veneer foamed aluminum composite [22]. Wei et al. used the response surface method and genetic algorithm to carry out lightweight design of the rear high floor skeleton assembly of the bus body, and adjusted the original model by referring to the three candidate points generated by the genetic algorithm and combined with the actual production, and the weight of the optimized high floor skeleton was reduced by 16% [23].

From the above, it can be seen that domestic and foreign research on lightweight frame structures mainly focuses on commercial vehicles, while research on the frame structure of mobile pump trucks that play an important role in emergency rescue has almost not been involved. Based on this, this paper addresses the original frame of a mobile pump truck. Considering the displacement, strength and weight reduction factors of the frame, the calculation analysis and lightweight optimization design of the longitudinal beam structure of the frame are carried out.

## 2 Frame model and calculation method

**Fig 1:**
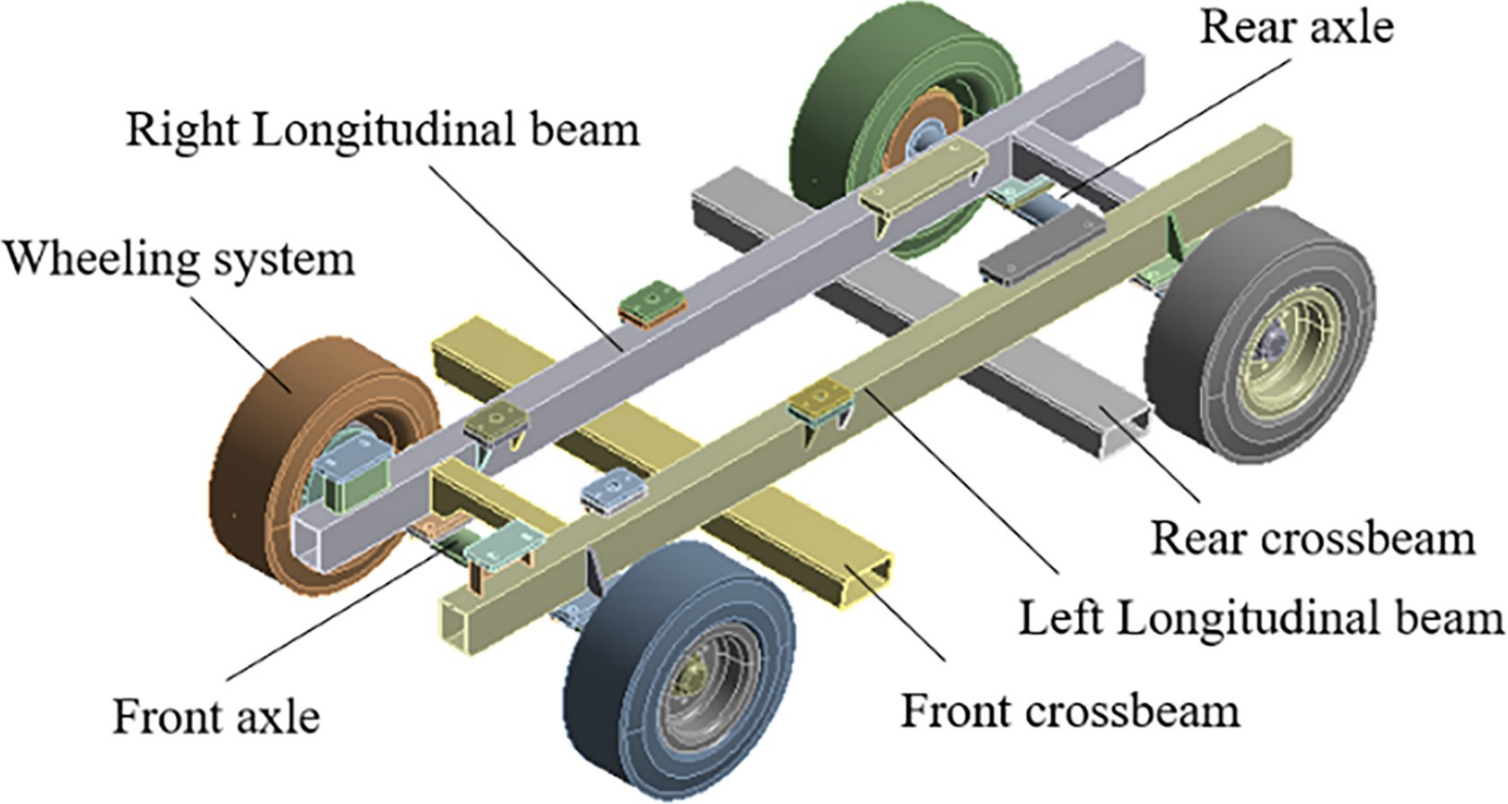
3D solid model of vehicle body.

The frame of the mobile pump truck mainly includes beams, axles, wheels and other connecting parts. The three-dimensional solid model of the car body is shown in Fig 1. Among them, the beam of the frame is made of Q345 low alloy structural steel; the axle, axle bracket and support plate are made of 40Cr; the tire material is made of automotive rubber; other parts are made of Q235 carbon structural steel. The material parameters are shown in Table 1.

**Table 1:** Frame material parameters.

Name	Materials	Density/(g·cm^-3^)	Poisson’s ratio	Elastic Modulus/GPa	Yield strength /MPa
Beam	Q345	7.85	0.2	206	345
Axles, axle supports, Support plates	40Cr	7.85	0.3	211	785
Tires	Rubber	1.2	0.47	7.8×10^−3^	-
Other parts	Q235	7.85	0.3	210	235

**Fig 2:**
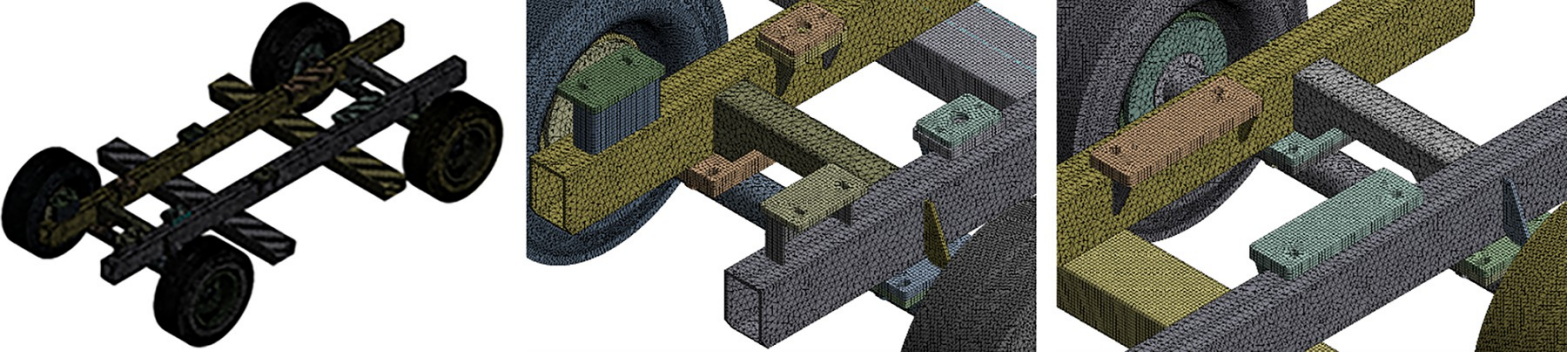
Meshing of vehicle body. (a) Overall meshing, (b) Local meshing of the front axle, (c) Local meshing of the rear axle.

According to the characteristics of the car body model, the local encryption method is adopted in this paper to improve the calculation accuracy, and the car body is divided into 612314 units and 1386074 nodes. The finite element model after grid division is shown in Fig 2.

## 3 Optimized design based on response surface

**Fig 3:**
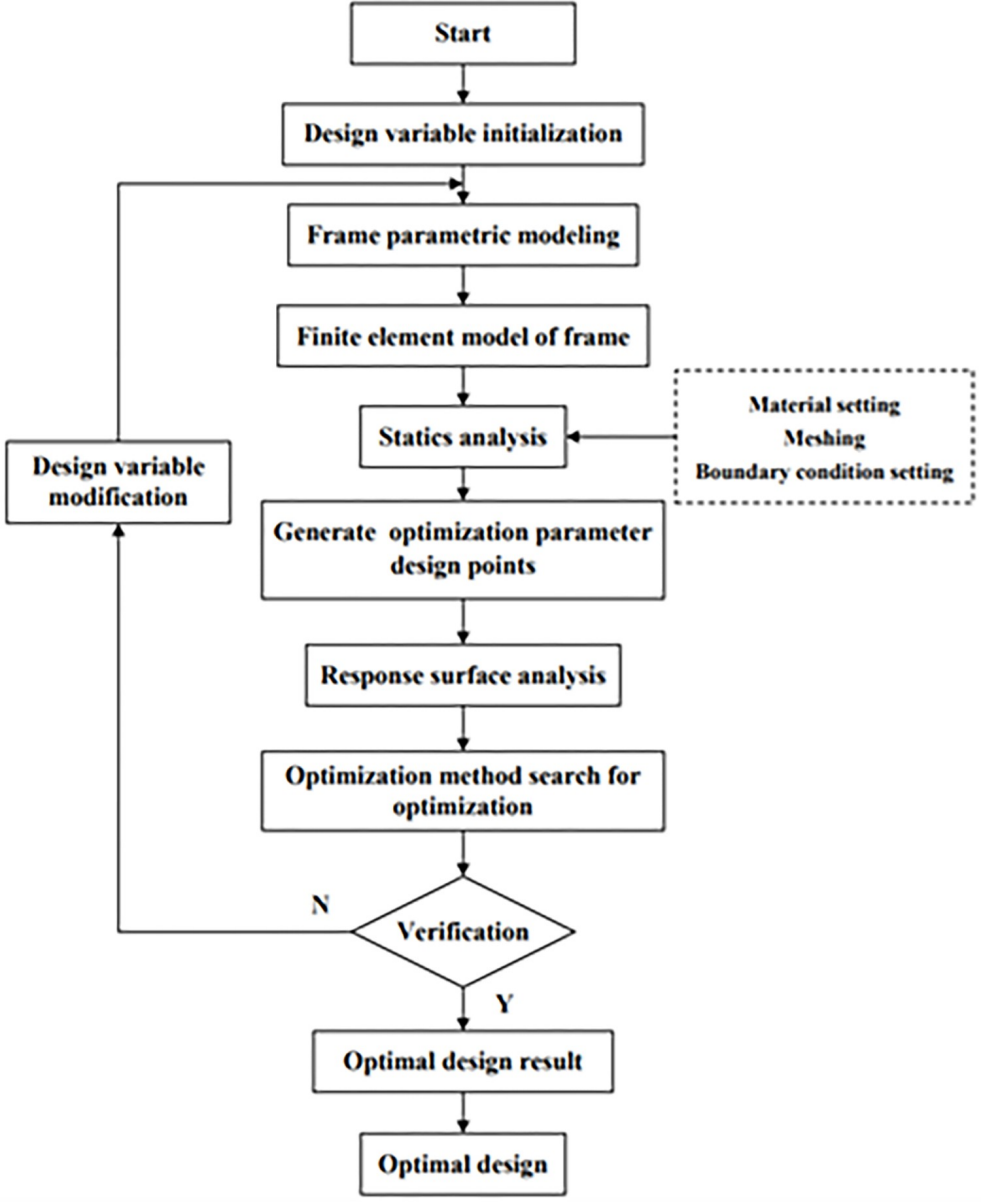
Optimization of the design process.

In order to achieve the goal of reducing the weight of the frame while ensuring its strength design requirements, the lightweight design goal of the frame is achieved. This article uses Response Surface Optimization of Ansys Workbench to optimize the design of the longitudinal beam structure, that is, to optimize its cross-sectional shape when the length of the longitudinal beam is determined. Based on the response surface method and MOGA (multi-objective genetic) optimization algorithm, this paper optimizes the structural parameters of the longitudinal beam of the frame. Through optimization analysis, multiple sets of optimization parameter design points are obtained and the frame optimization model is re-established for numerical calculation. The response surface analysis was carried out on the obtained data of each group, from which the optimal design scheme meeting the requirements of optimal design was obtained. The optimization design flow chart is shown in Fig 3.

### 3.1 Optimization of mathematical models

The purpose of this study is to reduce the weight of the frame by optimizing the shape of the longitudinal beam of the frame under the condition that the strength of the frame meets the requirements of the actual working conditions. In daily production and life, the typical working conditions of the mobile pump truck are mainly divided into four kinds: full load bending, full load torsion, emergency turning and emergency braking. Full load bending condition refers to when the mobile pump truck is driving on the flat road, the pump truck wheel and the road always maintain a stable contact, at this time the main consideration of the mobile pump truck in the vertical direction of the force. This is also the most common working state of the mobile pump truck, so this optimization will take the mass, maximum displacement and maximum stress of the frame under bending conditions as the objective function.

$$\min F(x)={\left\{F_{1}(x),F_{2}(x),F_{3}(x)\right\}}^{T}$$ (1)

$$\left\{ \begin{array}{l} F_{1}(x)=\min(m_{\max})\\ F_{2}(x)=\min(\omega_{\max})\\ F_{3}(x)=\min(\sigma_{\max}) \end{array}\right.$$ (2)

where: *m*_max_ is the mass of the frame, kg; *ω*_max_ is the maximum displacement of the frame, mm; *σ*_max_ is the maximum stress of the frame, MPa.

**Fig 4:**
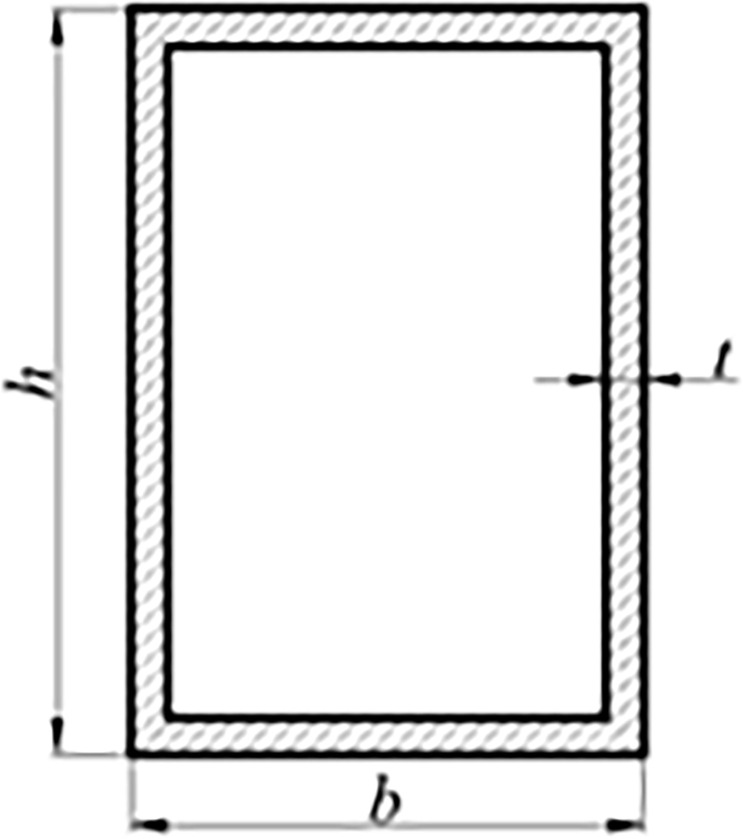
Shape of longitudinal beam section.

The response surface analysis method is used for multi-objective optimization. The height *h*, width *b* and thickness *t* of the longitudinal beam are taken as design variables. The value ranges of the design variables is shown in Table 2, and the cross-section shape of the longitudinal beam is shown in Fig 4.

**Table 2:** Value range of design variables.

Design variable	Initial value	Value range
Height *h*/mm	160	140~180
Width *b*/mm	110	90~130
Thickness *t*/mm	8	6~10

In summary, the optimization model for the multi-objective optimization problem of the longitudinal beam is established as follow:

$$\left\{ \begin{array}{l} \min F(x)={\left\{F_{1}(x),F_{2}(x),F_{3}(x)\right\}}^{T}\\ s.t.\left\{ \begin{array}{l} 140\leq h\leq 180\\ 90\leq b\leq 130\\ 6\leq t\leq 10 \end{array}\right.\\ X={\left[h,b,t\right]}^{T} \end{array}\right.$$ (3)

### 3.2 Optimization methods

The central composite method is selected for the design and 15 sets of design points are generated accordingly. The set mathematical model sets the constraints for calculation, and the data after the calculation is completed is shown in Table 3.

**Table 3:** Calculation results of optimized design points.

Order	Height *h*/mm	Width *b*/mm	Thickness *t*/mm	Maximum displacement *ω*_max_/mm	Maximum stress *σ*_max_/MPa	Frame weight *m*_max_/kg
1	160.0	110.0	8.0	2.1186	69.828	1159.6
2	144.0	110.0	8.0	2.1858	69.355	1146.3
3	176.0	110.0	8.0	2.0776	69.204	1172.9
4	160.0	99.0	8.0	2.1311	69.169	1150.4
5	160.0	121.0	8.0	2.1089	69.4	1168.8
6	160.0	110.0	7.2	2.1277	68.377	1139.5
7	160.0	110.0	8.8	2.1137	70.46	1179.4
8	146.99	101.06	7.3496	2.197	68.777	1126.5
9	173.01	101.06	7.3496	2.0968	68.725	1146.4
10	146.99	118.94	7.3496	2.1692	70.38	1140.2
11	173.01	118.94	7.3496	2.0804	69.896	1160.1
12	146.99	101.06	8.6504	2.175	69.768	1156
13	173.01	101.06	8.6504	2.0886	70.085	1179.4
14	146.99	118.94	8.6504	2.1531	70.102	1172.1
15	173.01	118.94	8.6504	2.0775	70.373	1195.5

**Fig 5:**
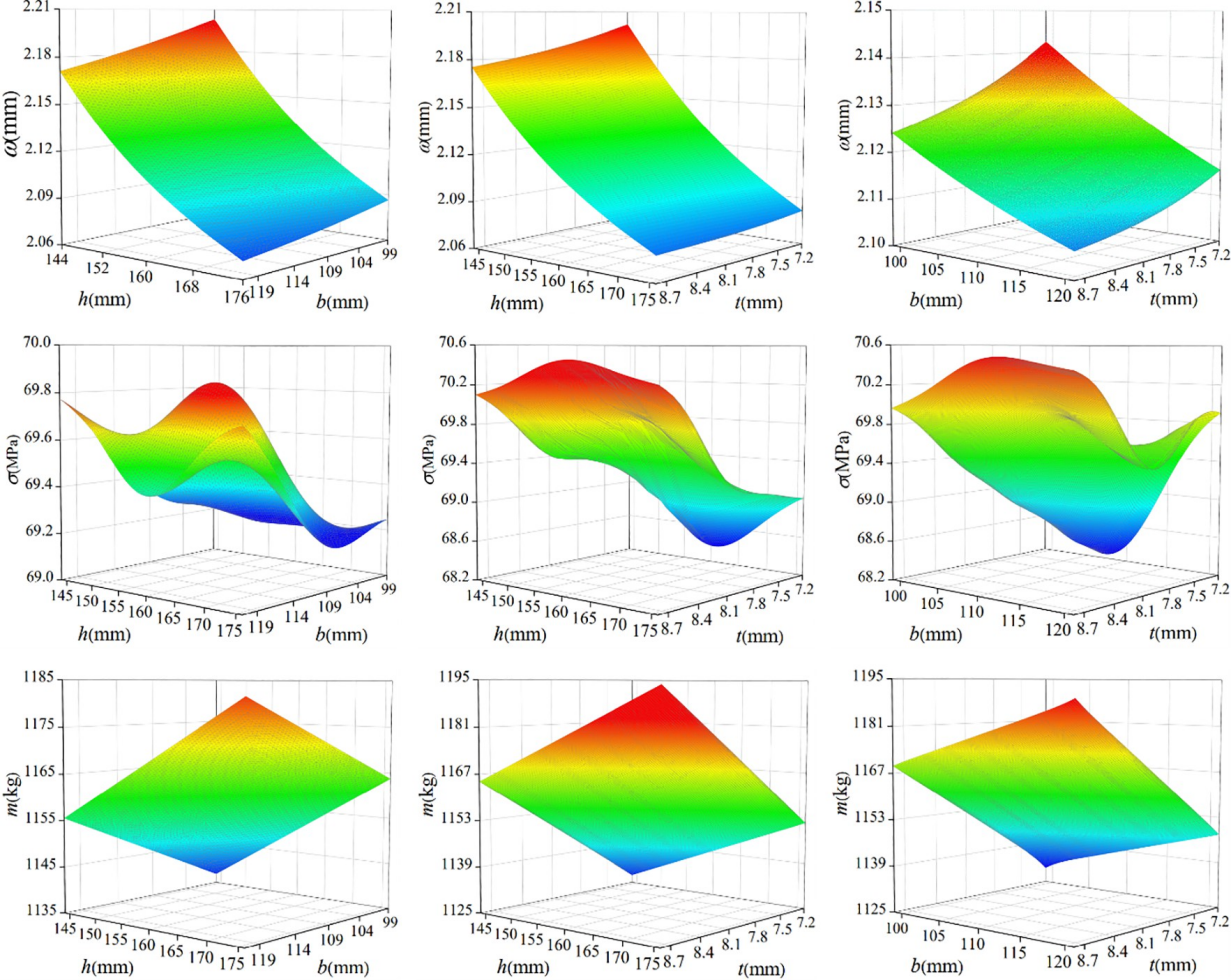
Response surface graph of lightweight model. (a) *h*-*b* displacement response surface (b) *h*-*t* displacement response surface (c) *b*-*t* displacement response surface (d) *h*-*b* stress response surface (e) *h*-*t* stress response surface (f) *b*-*t* stress response surface (g) *h*-*b* weight response surface (h) *h*-*t* weight response surface (i) *b*-*t* weight response surface.

The Genetic Aggregation algorithm is used to fit the response surface, and the response surface of input parameters to the output parameters (maximum displacement, maximum stress, frame weight) is generated. At the same time, MOGA(multi-objective genetic optimization algorithm) is used to solve the optimal parameters of the longitudinal beams. The maximum allowable Pareto percentage in the genetic algorithm is set to 70%, which is the convergence criterion (at least 70% of the samples in the population are included in the Pareto obtained in this iteration, and the iteration stops at the optimization frontier). The total number of initial samples is set as 3000, and three optimal design points are finally selected from the results. The response surface is shown in Fig 5, and the parameters of the candidate optimal design points are shown in Table 4.

**Table 4:** Candidate optimal design points.

Order	Height *h*/mm	Width *b*/mm	Thickness *t*/mm	Maximum displacement *ω*_max_/mm	Maximum stress *σ*_max_/MPa	Frame weight *m*_max_/kg
1	149.64	99.03	7.2023	2.19	68.814	1123.6
2	149.71	99.031	7.2014	2.1897	68.813	1123.6
3	149.61	99.208	7.2022	2.1899	68.806	1123.7

By comparing the above three candidates, the optimized dimensions are rounded. The optimized parameters are obtained as follows: the height of the longitudinal beam *h* is 150mm, the width of the longitudinal beam *b* is 99mm, and the thickness of the longitudinal beam *t* is 7.2mm. Compared to the weight of the frame before optimization, the weight of the optimized frame has been reduced by 35.8kg. The parameter changes are shown in Table 5.

**Table 5:** Parameter changes before and after optimization.

Item	Height *h*/mm	Width *b*/mm	Thickness *t*/mm	Frame weight *m*_max_/kg
Original	160	110	8	1159.6
Optimized	150	99	7.2	1123.8
Difference	10	11	0.8	35.8

## 4 Optimization analysis result

### 4.1 Boundary condition setting

According to the optimization analysis, the new structural parameters of the longitudinal beam are obtained, and the numerical simulation analysis is carried out on the frame of the mobile pump truck before and after optimization. In this paper, the finite element analysis software of Ansys Workbench is used to conduct simulation experiments from four working conditions of the frame: full-load bending, full-load torsion, emergency turning and emergency braking, and to compare and analyze the changes of performance parameters before and after optimization. When the mobile pump truck is driving stably on the flat road, the working condition is full-load bending condition, in which the pump truck is mainly subjected to the vertical force, so all the degrees of freedom of the front and rear four wheels are constrained when the boundary conditions are set. Full load torsion condition refers to the situation that a wheel of the moving pump suddenly appears in the process of driving. This condition will lead to the asymmetric support of the frame, so all the freedom of the left front wheel is released when the boundary condition is set, all the freedom of the right front wheel is restricted, and the freedom of the vertical direction of the two rear wheels is restricted, so as to simulate the situation of the left front wheel suddenly hanging in the driving process of the mobile pump truck. When the mobile pump truck makes an emergency turn, the displacement and stress of the pump truck frame will change due to the centripetal acceleration. In this paper, 0.4g left-side acceleration is applied to the frame to simulate the state change of the frame under turning conditions, and the translational freedom of the front and rear four wheels in three directions XYZ is constrained, while the rotation freedom of the four wheels in three directions XYZ is released. When the mobile pump truck is subjected to emergency braking, the displacement and stress of the frame will change due to the longitudinal inertial force load. Therefore, 0.45g deceleration acceleration is applied to the frame in this paper to simulate the state change of the frame under braking conditions. The translational freedom of the front wheel in three directions XYZ is constrained when boundary conditions are set, and the rotational freedom of the three directions XYZ is released. Constrains the vertical degrees of freedom UY and vertical degrees of freedom UZ of the rear wheel, freeing the other degrees of freedom. In addition, the weight of the diesel engine and self-priming pump on the mobile pump is 600kg and 500kg respectively. The setting of boundary conditions of each working condition of the frame is shown in Table 6. (transverse: X, vertical: Y, longitudinal: Z, U is the translational degree of freedom, ROT is the rotational degree of freedom, the rest of the working conditions are the same).

**Table 6:** Setting of boundary conditions for each working condition.

Work conditions	Left front wheel	Right front wheel	Left rear wheel	Right rear wheel
Full load bending	All	All	All	All
Full load reversal	-	All	UY	UY
Emergency turns	UXUYUZ	UXUYUZ	UXUYUZ	UXUYUZ
Emergency braking	UXUYUZ	UXUYUZ	UYUZ	UYUZ

**Fig 6:**
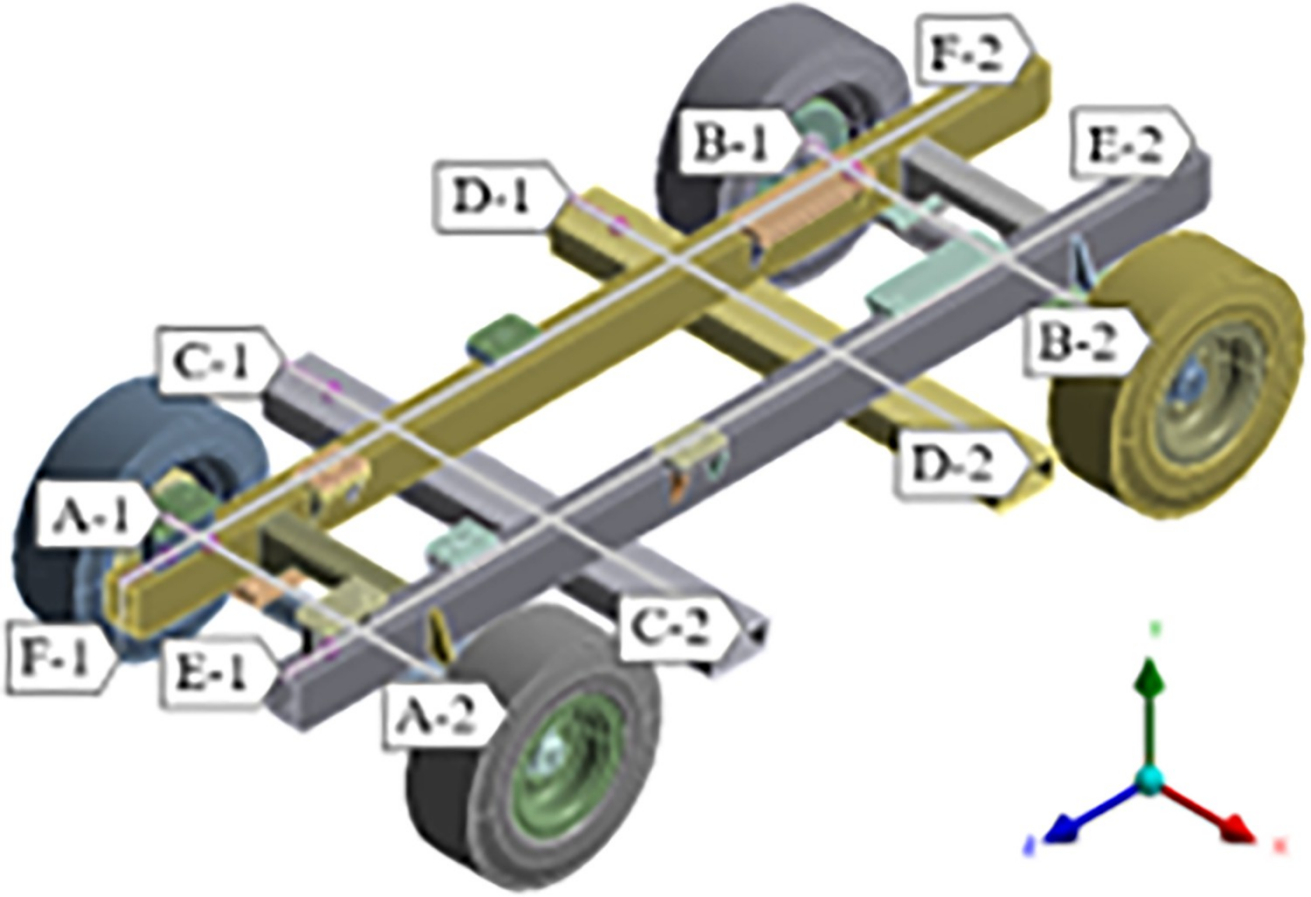
Schematic diagram of the monitoring path.

In this paper, a monitoring path is added at the center line of the upper surface of the front and rear axles, front and rear beams and left and right longitudinal beams of the frame. The six components are all hollow structures, and the monitoring path is from the starting point (1) to the end point (2). The Schematic diagram of the monitoring path is shown in Fig 6.

### 4.2. Full load bending condition performance comparison

**Fig 7:**
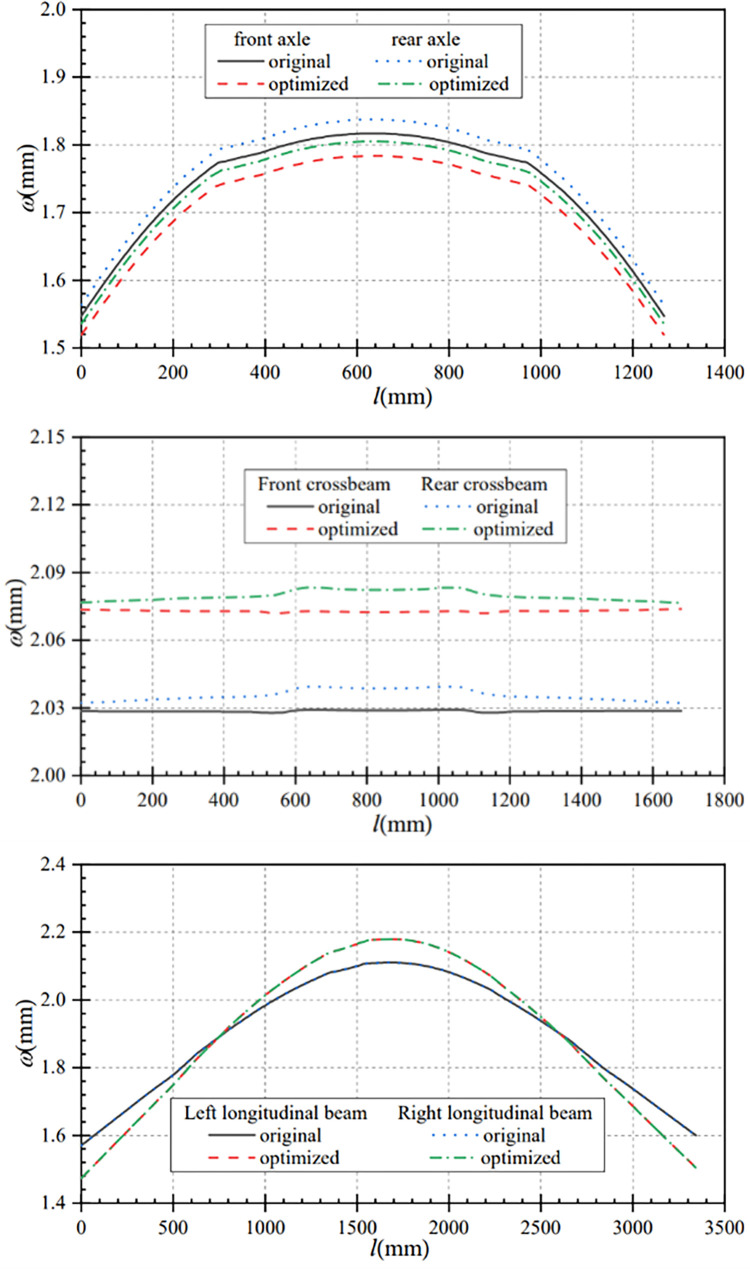
Displacement comparison curves of main components under bending condition. (a) Contrast curve of axle displacement (b) Contrast curve of crossbeam displacement (c) Contrast curve of longitudinal beam displacement.

In this paper, the data before and after the optimization of the longitudinal beam section are compared and analyzed, so as to get the displacement comparison curves and stress comparison curves of the main components under bending conditions. From Fig 7(A), it can be seen that the maximum axle displacement after optimization is smaller than that before optimization under the bending condition. The maximum displacement of front and rear axles before optimization are 1.82mm and 1.84mm respectively, and the maximum displacement of front and rear axles after optimization are 1.78mm and 1.81mm respectively. The displacements change are 0.04mm and 0.03mm respectively. That is, the optimized displacements of front and rear axles are reduced by 2.20% and 1.63% separately.

Fig 7(B) shows the comparison curve of crossbeams displacement before and after optimization. The overall displacement of the front and rear crossbeams after optimization is slightly larger than that before optimization. The maximum displacement of front and rear crossbeam increased from 2.03mm and 2.04mm before optimization to 2.07mm and 2.08mm after optimization separately, and the displacement changes are 0.04mm. That is, the displacement of front and rear crossbeam increased by 1.97% and 1.96% respectively after optimization. It can be seen that the increase of axle displacement after optimization is smaller than that before optimization.

From Fig 7(C), it can be seen that the displacements at both ends of the optimized longitudinal beam are smaller than that before optimization. The two end displacements of the longitudinal beam before optimization are 1.57mm and 1.60mm, and the two end displacements after optimization are 1.47mm and 1.51mm. The maximum displacement of the optimized longitudinal beams is 2.18mm, which is 0.07mm higher than that before optimization. That is, the maximum displacement of the optimized longitudinal beams is increased by 3.31%. Under the bending condition, the displacement changes of the main components before and after optimization are less than 0.1mm, and the variation range is small.

**Fig 8:**
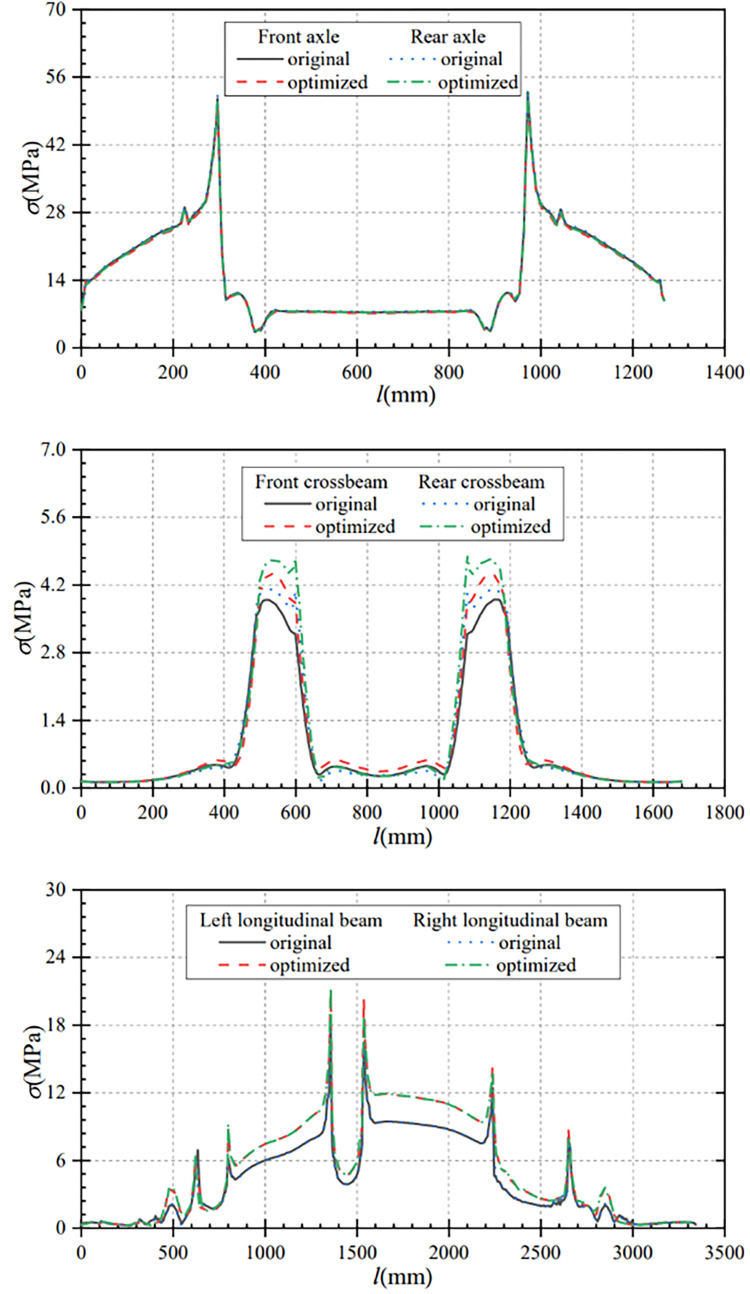
Stress comparison curves of main components under bending conditions. (a) Contrast curve of axle stress (b) Contrast curve of crossbeam stress (c) Comparison curve of longitudinal beam stress.

As can be seen from Fig 8(A), there is no significant change in the stress and stress variation trend of the optimized axle compared with that before optimization under bending conditions. The maximum stress of the optimized front axle is 51.71MPa, which is reduced by 1.10MPa compared with that before optimization. The maximum stress of the optimized rear axle is 52.39MPa, which is reduced by 1.09MPa compared with that before optimization. That is, the stresses of the optimized front and rear axles are reduced by 2.08% and 2.03% respectively.

Fig 8(B) shows the comparison curves of stress changes in the crossbemas before and after optimization. The stress change trend of the front and rear crossbeams after optimization is similar to that before optimization. Compared with the pre-optimization, the maximum stress of the optimized crossbeam is increased, the maximum stress of the front crossbeam is increased from 3.90MPa to 4.43MPa, and the stress variation is 0.53MPa. The maximum stress of the rear crossbeam increases from 4.11MPa to 4.83MPa with a stress change of 0.72MPa, which is a smaller change.

Fig 8(C) shows the comparison curves of stress changes in the longitudinal beams before and after optimization. The overall stress variation trend of the longitudinal beams before and after optimization is roughly the same, but the stress of the longitudinal beam after optimization is larger than that before optimization. The maximum stress of the left longitudinal beam after optimization is 20.85MPa, which is 3.16MPa higher than that before optimizion; the maximum stress of the right longitudinal beam after optimization is 21.37 MPa, which is 3.58 MPa higher than that before optimization. Under the bending condition, the stress variation of the main components before and after optimization is less than 4MPa, and the variation range is small. At the same time, the stress of the optimized frame is much less than the allowable stress of the material.

### 4.3 Full load torsional performance comparison

**Fig 9:**
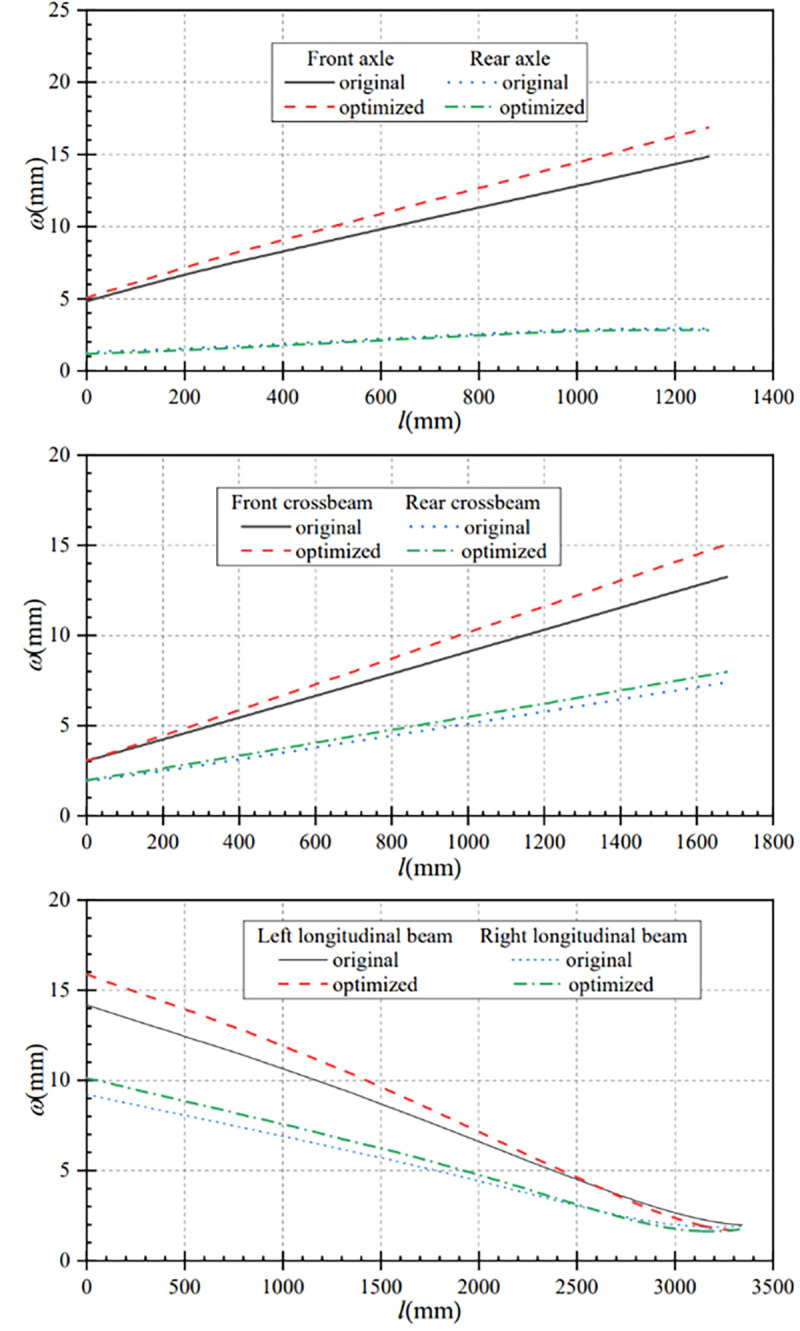
Displacement comparison curves of main components under torsion condition. (a) Contrast curve of axle displacement (b) Contrast curve of crossbeam displacement (c) Contrast curve of longitudinal beam displacement.

In this paper, the data under torsion condition before and after optimization are compared and analyzed, so as to obtain the displacement contrast curve of the main components and the stress contrast curve of the main components under this condition. Fig 9(A) shows the comparison curves of the axle displacement changes before and after optimization under torsional conditions. Under this condition, the optimized front axle displacement is increased, and the rear axle displacement is approximately the same as that before optimization. The maximum displacement of the optimized front axle is 16.87mm, which is 2.02mm larger than that before optimization.

Fig 9(B) shows the comparison curves of the crossbeams displacement changes before and after optimization under this working condition. Compared with before optimization, the displacements of the front and rear crossbeams have increased to some extent after optimization. The maximum displacement of the front crossbeam after optimization is 15.06mm, which is 1.80mm higher than that before optimization; the maximum displacement of the rear crossbeam after optimization is 7.98mm, which is 0.58mm higher than that before optimization.

From Fig 9(C), it can be seen that the maximum displacement of the optimized left longitudinal beam is 15.88mm, which is 1.70mm larger than that before optimization. And from *l* = 2632mm, the displacement of the left longitudinal beam after optimization is smaller than that before optimization. Similarly, the maximum displacement of the optimized right longitudinal beam is 10.13mm, which is 0.91mm larger than that before optimization. And starting from *l* = 2603mm, the displacement of the optimized left longitudinal beam is smaller than that before optimization.

**Fig 10:**
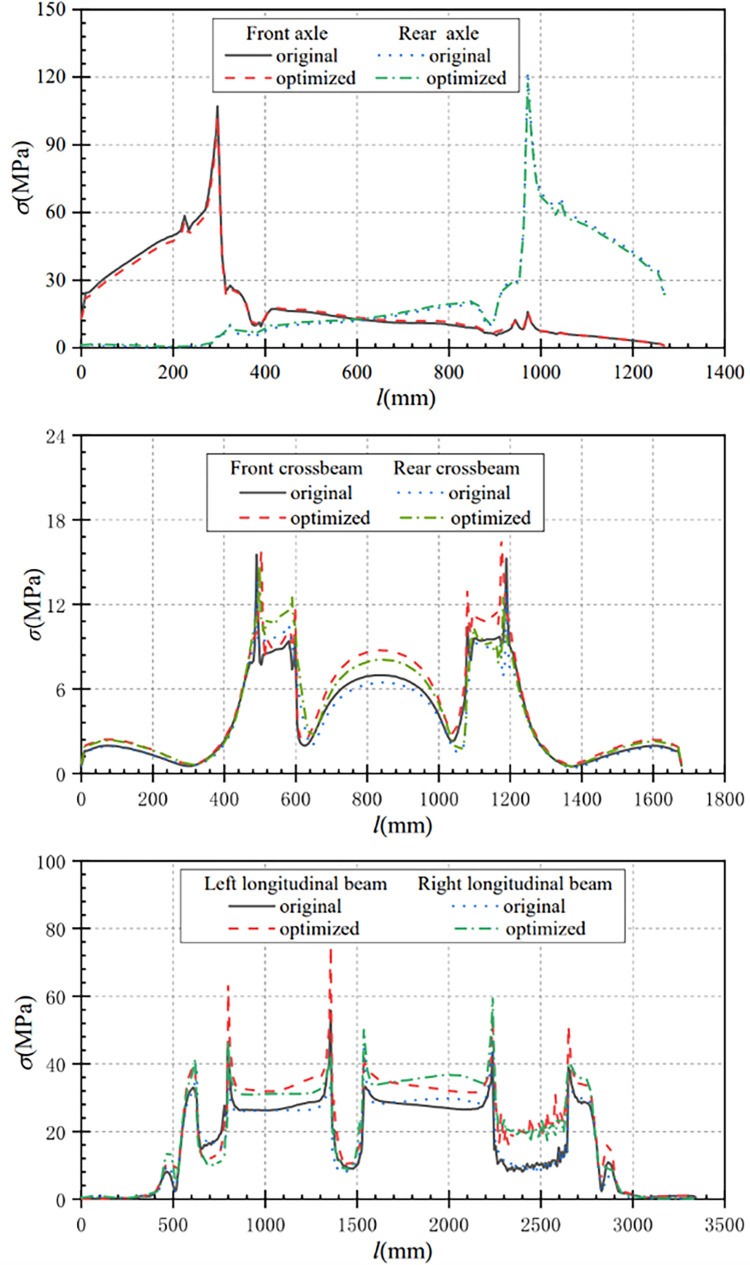
Stress comparison curves of main components under torsion condition. (a) Contrast curve of axle stress (b) Contrast curve of crossbeam stress (c) Comparison curve of longitudinal beam stress.

From Fig 10(A), it can be seen that the maximum stress of the optimized front axle is 102.39MPa under torsional conditions, which is 4.65MPa less than the maximum stress of the front axle before optimization. The maximum stress in the rear axle after optimization is 118.79 MPa, which is 3.66 MPa less than that before optimization. The maximum stress in the front and rear axles after optimization is reduced by 4.34% and 2.99% separately.

From Fig 10(B), it can be seen that the maximum stress of the optimized crossbeam increased compared with that before optimization. The maximum stress of the front crossbeam increased from 15.53MPa before optimization to 16.59MPa after optimization, with a stress change of 1.06Mpa. The maximum stress of the rear crossbeam is increased from 13.71MPa before optimization to 14.75MPa after optimization, with a stress change of 1.04MPa. The stress variation range before and after the crossbeam optimization is small.

From Fig 10(C), it can be seen that the overall stress change trend of the longitudinal beam before and after optimization is approximately the same. After optimization, the maximum stress of the left longitudinal beam after optimization is 74.73MPa, which is increased by 18.99MPa compared with that before optimization. Similarly, the maximum stress of the right longitudinal beam after optimization is 60.16MPa, which is increased by 12.39MPa compared with that before optimization. Compared with before optimization, the optimized frame stress is increased to a certain extent, but the stress is far less than the yield strength limit of the material, and the optimized frame still meets the strength design requirements. However, in the actual production process, it should still try to avoid reversing the situation.

### 4.4 Performance comparison of emergency turning conditions

**Fig 11:**
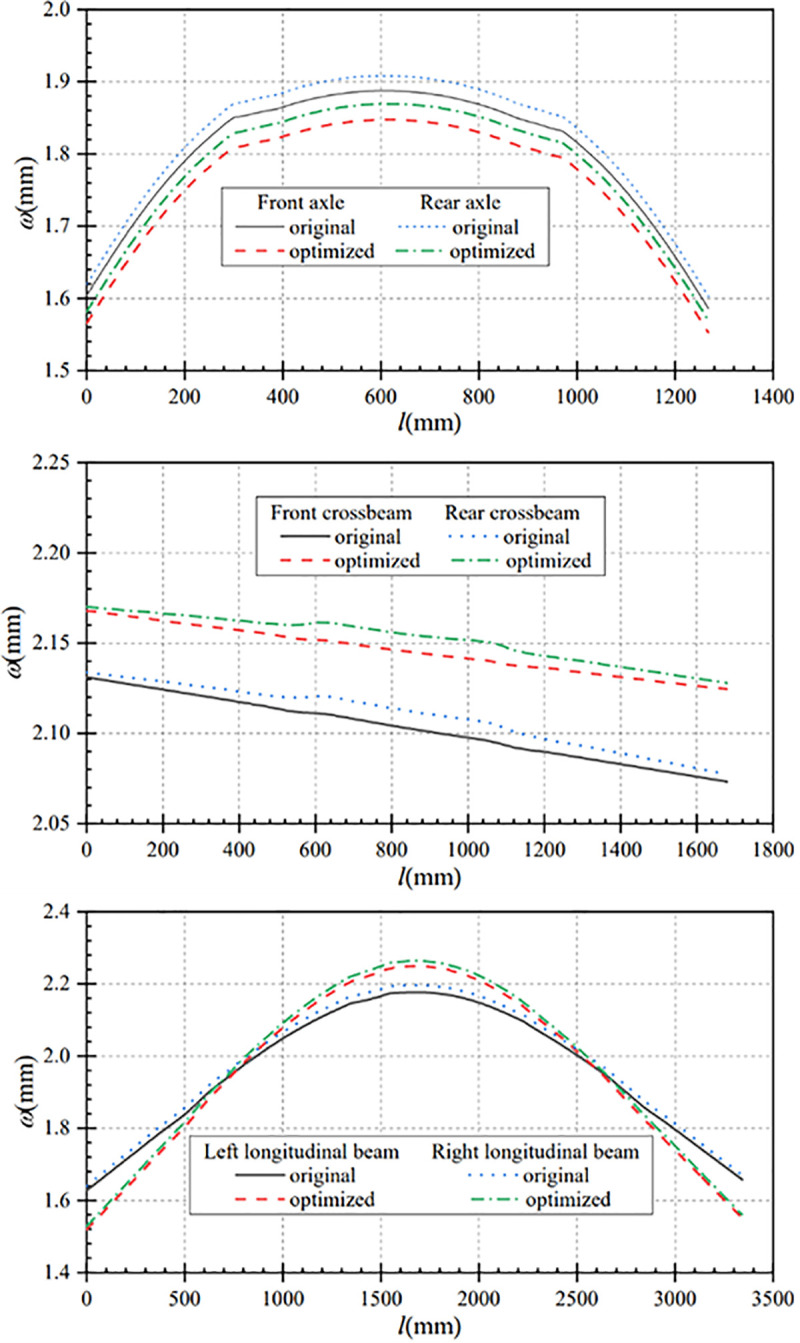
Displacement comparison curves of main components under the turning condition. (a) Contrast curve of axle displacement (b) Contrast curve of crossbeam displacement (c) Contrast curve of longitudinal beam displacement.

In this paper, the data under turning condition before and after optimization are compared and analyzed, so as to obtain the displacement comparison curves and stress comparison curves of the main components under this condition. Fig 11(A) shows the comparison curves of axle displacement changes before and after optimization. In the turning condition, the maximum axle displacement after optimization is smaller than that before optimization. The maximum displacement of the front axle is reduced from 1.89 mm before optimization to 1.85mm after optimization, and the maximum displacement of the rear axle is reduced from 1.91mm to 1.87mm, both of which decreased by 0.04 mm. That is, the maximum displacement of the front and rear axles is reduced by 2.12% and 2.09% respectively.

Fig 11(B) shows that the overall displacement of the front and rear crossbeams after optimization is slightly larger than the overall displacement before optimization, and the change trend of crossbeam displacement before and after optimization is approximately the same. The maximum displacement of the front beam increased from 2.13mm before optimization to 2.17mm after optimization, with a displacement change of 0.04mm. The maximum displacement of the rear crossbeam is increased from 2.13mm before optimization to 2.17 mm after optimization, with a displacement change of 0.04mm. After optimization, the maximum displacement of the front and rear beams increases by 1.88%, and the variation range is small.

As can be seen from Fig 11(C), it can be seen that the displacement at both ends of the optimized longitudinal beams is smaller than that before optimization, and the maximum displacement of the optimized longitudinal beam is larger than that before optimization. The front-end displacement of the left longitudinal beam after optimization is 1.52mm, which is reduced by 0.11mm compared with that before optimization; the maximum displacement after optimization is 2.25mm, which is increased by 0.07mm compared with that before optimization. After optimization, the back-end displacement is 1.56mm, which is reduced by 0.11mm compared with that before optimization. Similarly, the front-end displacement of the right longitudinal beam after optimization is 1.53mm, which is reduced by 0.10mm compared with that before optimizationless; the maximum displacement after optimization is 2.26mm, which is increased by 0.07mm compared with that before optimization. After optimization, the back-end displacement is 1.56mm, which is reduced by 0.11mm compared with that before optimization. In the turning condition, the overall displacement of the optimized frame and the displacement of each key component are smaller.

**Fig 12:**
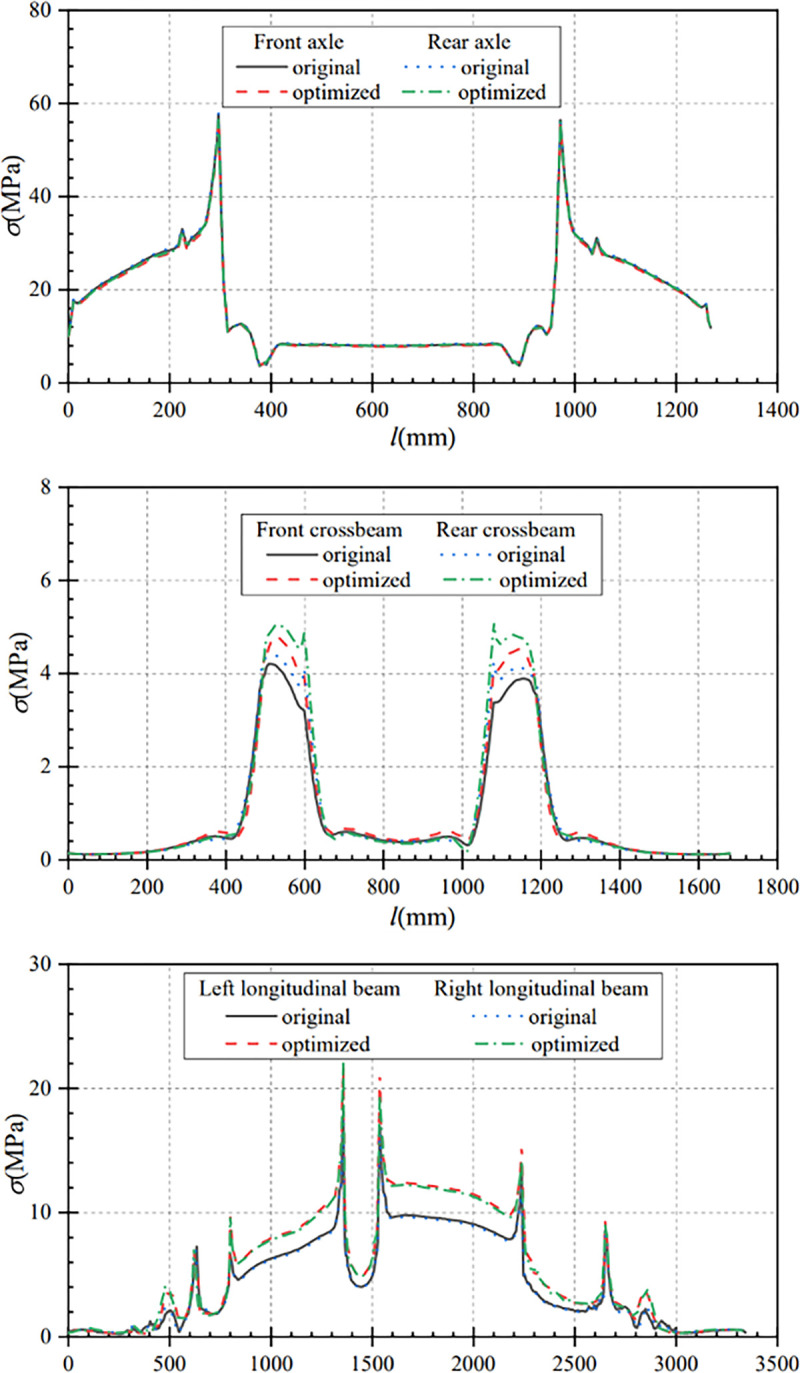
Stress comparison curves of main components under the turning condition. (a) Contrast curve of axle stress (b) Contrast curve of crossbeam stress (c) Comparison curve of longitudinal beam stress.

As can be seen from Fig 12(A), the axle stress and stress change trends are approximately equal before and after optimization. The maximum stress of the optimizated front axle is 56.25MPa, which is reduced by 0.96MPa compared with that before optimization. After optimization, the maximum stress of the rear axle is 56.88MPa, which is reduced by 1.38MPa compared with that before optimization. That is, the maximum stress of the optimized front and rear axles is reduced by 1.68% and 2.36% respectively.

Fig 12(B) shows that the maximum stress of the optimized crossbeam is increased. Compared with before optimization, the maximum stress of the front crossbeam is increased from 4.21MPa before optimization to 4.76MPa after optimization, and the stress variation is 0.55MPa. The maximum stress of the rear crossbeam is increased from 4.39MPa before optimization to 5.10MPa after optimization, and the stress variation is 0.71 MPa, which is a small change in stress.

It can be seen from Fig 12(C) that the overall stress change trend of the longitudinal beam before and after optimization is approximately the same, but the stress of the longitudinal beam after optimization is larger than that before optimization. After optimization, the maximum stress of the left longitudinal beam is 21.74MPa, which is increased by 3.46MPa compared with that before optimization; and the maximum stress of the right longitudinal beam after optimization is 22.00MPa, which is increased by 3.83MPa compared with that before optimization. The stress of the optimized frame is much less than the allowable stress of the material, so the optimized frame meets the requirements of strength design.

### 4.5 Emergency braking conditions performance comparison

**Fig 13:**
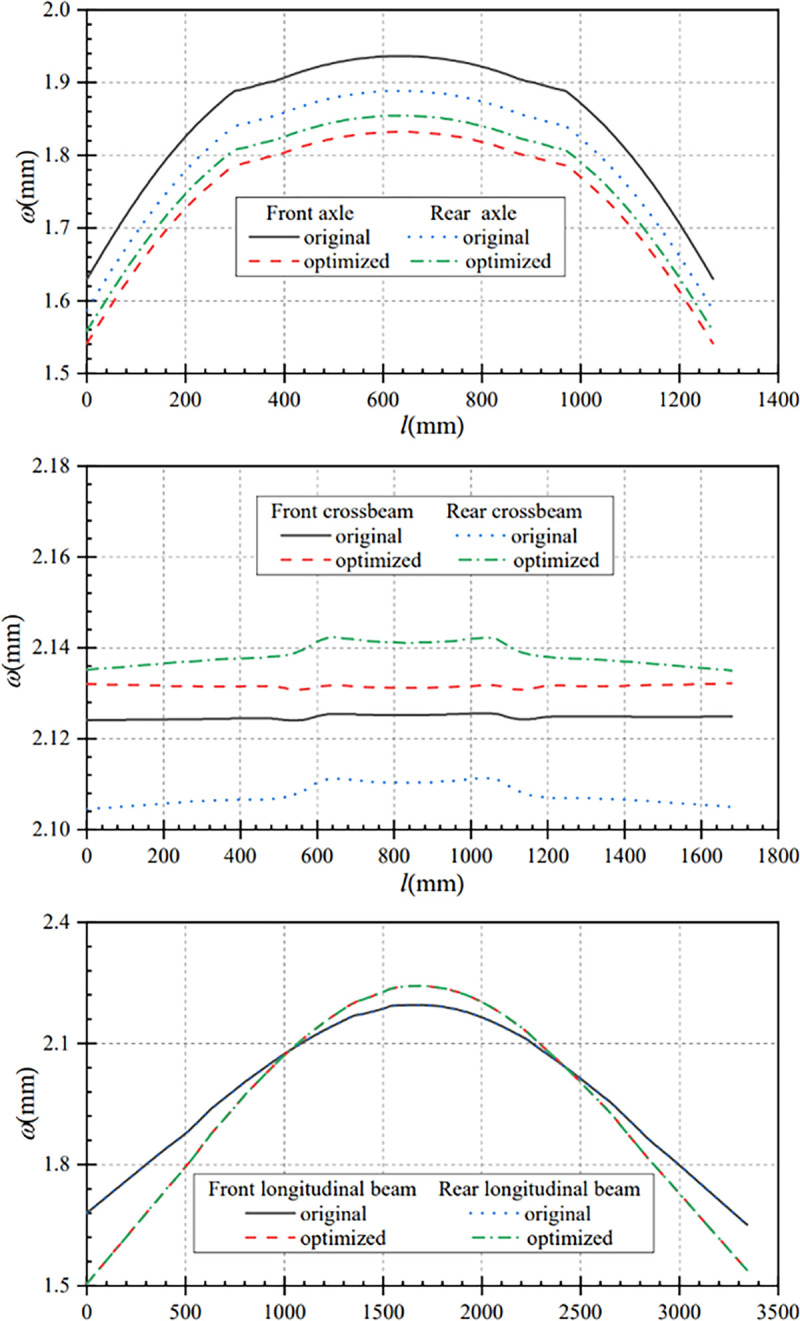
Displacement comparison curves of main components under braking condition. (a) Contrast curve of axle displacement (b) Contrast curve of crossbeam displacement (c) Contrast curve of longitudinal beam displacement.

In this paper, the data under the braking condition before and after optimization are compared and analyzed, so as to obtain the main component displacement comparison curve and main component stress comparison curve under this working condition. From Fig 13(A), it can be seen that the maximum displacement of the axle after optimization is smaller than that before optimization under the braking condition. The maximum displacement of the front axle is reduced from 1.94mm before optimization to 1.83mm after optimization, with a displacement change of 0.11mm; the maximum displacement of the rear axle is reduced from 1.89mm before optimization to 1.85mm after optimization, with a displacement change of 0.04mm. That is, the maximum displacement of the optimizated front and rear axles is reduced by 5.67% and 2.12% respectively.

Fig 13(B) shows the crossbeam displacement comparison curves before and after optimization, the overall displacement of the front and rear crossbeams after optimization is slightly larger than the overall displacement before optimization. The maximum displacement of the front crossbeam is increased from 2.125mm before optimization to 2.132mm after optimization, with a displacement change of 0.007mm. The maximum displacement of the rear crossbeam is increased from 2.112mm before optimization to 2.142mm after optimization, with a displacement change of 0.03mm. After optimization, the maximum displacement of the front and rear crossbeams are increased by 0.33% and 1.42%, and the displacement change is small.

As can be seen from Fig 13(C), the displacements of the ends of the optimized longitudinal beam are smaller than that before optimization. The displacements at both ends of the longitudinal beam are 1.68mm and 1.65mm before optimization, and 1.51mm and 1.54mm after optimization, which are reduced by 0.17mm and 0.11mm separately. Compared to the maximum displacement of 2.20mm of the longitudinal beam before optimization, the maximum displacement of the optimized longitudinal beam is increased by 0.04mm to 2.24mm. That is, the displacement of both ends of the optimized longitudinal beam is reduced by 10.11% and 6.67% respectively, while the maximum displacement of the longitudinal beam is increased by 1.82%. Under the braking condition, the displacement changes of the main components before and after optimization are less than 0.2mm, and the variation range is small.

**Fig 14:**
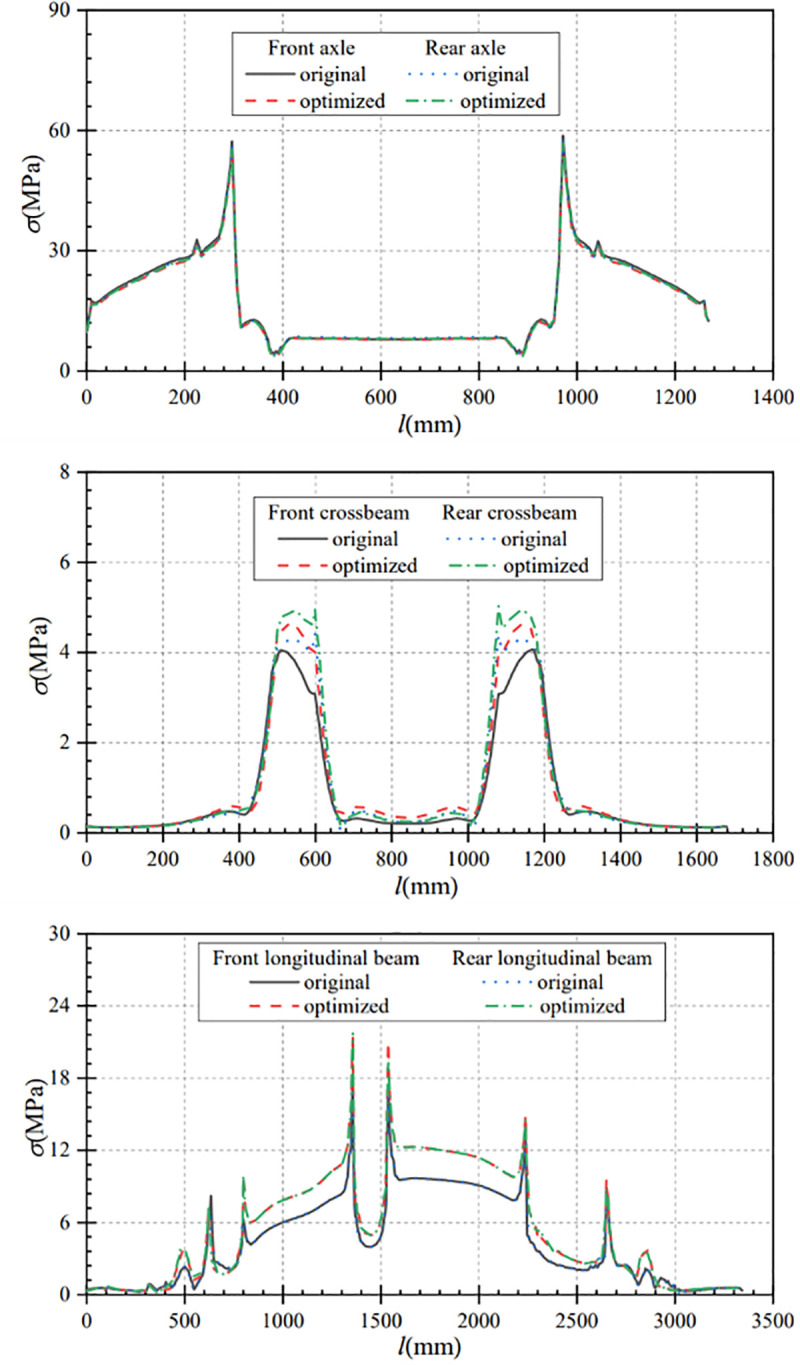
Stress comparison curves of main components under braking condition. (a) Contrast curve of axle stress (b) Contrast curve of crossbeam stress (c) Comparison curve of longitudinal beam stress.

From Fig 14(A), it can be seen that the axle stresses and stress variation trends before and after optimization are approximately equal under braking conditions. The optimized front axle maximum stress is 56.62MPa, which is 2.02MPa that before optimization. The optimized rear axle maximum stress is 57.31MPa, which is reduced by 0.16MPa compared with that before optimization. That is, the maximum stress of the optimized front and rear axles is reduced by 3.44% and 0.28% respectively.

As can be seen from Fig 14(B), the maximum stress of the front crossbeam increased from 4.06MPa before optimization to 4.64MPa after optimization, and the change of stress is 0.58MPa. The maximum stress of the rear crossbeam increases from 4.42MPa before optimization to 5.02MPa after optimization, and the stress change is 0.60MPa. The range of stress change before and after optimization is small.

As can be seen from Fig 14(C), the overall stress change trend of the longitudinal beam before and after optimization is approximately the same, but the stress of the longitudinal beam after optimization is larger than that before optimization. After optimization, the maximum stress of the left longitudinal beam after optimization is 21.60MPa, which is increased by 3.54MPa compared with that before optimization. The maximum stress of the right longitudinal beam after optimization is 22.13MPa, which is increased by 3.97MPa compared with that before optimization. Under braking conditions, the stress variation of the main components before and after optimization is less than 4MPa, and the stress variation range is small. At the same time, the stress of the optimized frame is much less than the allowable stress of the material.

## 5. Conclusion

In this paper, the longitudinal beam structure of the frame is redesigned, and the static characteristics of the frame of the mobile pump truck are compared and analyzed under four typical working conditions: full-load bending, full-load torsion, emergency turning and emergency braking. The main conclusions are as follows:

1. In the frame of the mobile pump truck, the materials used in each part can meet the strength design requirements of the frame structure and there is a certain optimization space. After optimization, the height of the longitudinal beam is reduced by 10mm, the width of the longitudinal beam is reduced by 11mm, the thickness of the longitudinal beam is reduced by 0.8mm, and the mass of the frame model is reduced by 35.8kg.
2. Under the three working conditions of full load bending, emergency turning and emergency braking, the maximum displacement of the optimized axle is smaller than the maximum displacement of the pre-optimized axle. The maximum displacement of the optimized front and rear crossbeams is slightly larger than the maximum displacement of the pre-optimized crossbeam. In addition, the displacement of both ends of the optimized longitudinal beam is smaller than that of the optimized front longitudinal beam, but the maximum displacement of the optimized longitudinal beam is larger than that of the optimized front longitudinal beam, and the increase amplitude is small.
3. Under the conditions of full load bending, emergency turning and emergency braking, the maximum stress of the optimized axle is reduced compared with that before optimization. After optimization, the crossbeam stress and the longitudinal beam stress are increased, but the increase is small.
